# Traditional Chinese-Hong Kong version of Forgotten Joint Score-12 (FJS-12) for patients with osteoarthritis of the knee underwent joint replacement surgery: cross-cultural and sub-cultural adaptation, and validation

**DOI:** 10.1186/s12891-022-05156-5

**Published:** 2022-03-08

**Authors:** Kevin Ki-Wai Ho, Wai-Wang Chau, Lawrence Chun-Man Lau, Michael Tim-Yun Ong

**Affiliations:** 1 Department of Orthopaedics and Traumatology, Chinese University of Hong Kong, Hong Kong SAR, China; 2 Department of Orthopaedics and Traumatology, Prince of Wales Hospital, Hong Kong SAR, China

**Keywords:** FJS-12, Total knee arthroplasty, Traditional Chinese-Hong Kong, Validation, Sub-cultural adaptation

## Abstract

**Background:**

A patient-reported outcome (PRO) tool which reflects the outcomes of patients underwent total knee arthroplasty (TKA) are important to be “ceiling effect free” which commonly used PRO tools face. Forgotten joint score-12 (FJS-12) has been proved to reduce or even free from ceiling effect. FJS-12 has been translated to different languages. The objectives of this study are to validate FJS-12 in Traditional Chinese-Hong Kong language and look for the goodness of FJS-12 still exist in this language adapted FJS-12 version.

**Methods:**

FJS-12 was administered to 75 patients whose majority was obese underwent TKA between September 2019 and March 2020. Patients completed 3 sets of questionnaires (FJS-12, Oxford Knee Score (OKS), and Numeric Rating Scale (NRS)) twice, 2 weeks apart. Reliability, internal consistency, responsiveness, test–retest agreement and discriminant validity were evaluated.

**Results:**

Reliability of FJS-12 showed moderate to excellent internal consistency (Cronbach’s *α* = 0.870). Test–retest reliability of FJS-12 was good (ICC = 0.769). Bland–Altman plot showed good test–retest agreement. Construct validity in terms of correlations between FJS-12 and OKS, and FJS-12 and NRS were moderate at baseline (Pearson’s coefficient *r* = 0.598) and good at follow-up (*r* = 0.879). Smallest detectable change (Responsiveness) was higher than MIC. Floor effect was none observed, and ceiling effect was low. Discriminant validity was found to have no significance. BMI (obesity) did not affect FJS-12 outcomes.

**Conclusions:**

The Traditional Chinese-Hong Kong version of FJS-12 showed good test–retest reliability, validity, responsiveness, BMI non-specific, with no floor and low ceiling effects for patients who underwent TKA. Sub-culture differences in individual PRO tools should be considered in certain ethnicities and languages.

**Supplementary Information:**

The online version contains supplementary material available at 10.1186/s12891-022-05156-5.

## Introduction

Using patient-reported outcome (PRO) aiming at measuring the health-related quality of life (HRQOL) of end stage knee arthritis patients underwent knee arthroplasty has been well received [1]. The use of PRO is proven useful to reflect and understand the HRQOL of the patients suffering from their disorder severity [1]. PRO also provides timely and appropriate therapeutic and rehabilitation strategies. The success of a disease-specific PRO always comes with their well cross-cultural adaptation capability which make them locality and language friendly [2].

Forgotten Joint Score-12 (FJS-12) is a newly developed well-recognized joint-specific patient-reported outcome (PRO) focusing on patients’ awareness of a specific joint in everyday life [3]. Joint awareness is always ‘forgotten’ until strong sensations come e.g. pain, mild stiffness, subjective dysfunction, or any discomfort [3]. FJS-12 has been introduced in different joint related studies [4–10] together with some "gold standards", such as Western Ontario and McMaster Universities Osteoarthritis Index (WOMAC) [11, 12], Oxford Knee Score (OKS) [13], Knee Injury and Osteoarthritis Outcome Score (KOOS) [14], Knee Society Score (KSS) and Function Score (KFS) [15]. Recent technology allows patients to look for the information concerning their disease symptoms, treatments receiving and expected outcomes. Gaining knowledge benefits the patients and at the same time, they expect better health outcomes as medical technology (knee arthroplasty) advances. Some of the tools mentioned before, as the PRO’s internal construct has been developed for years, find themselves difficult to differentiate between higher levels of function and patient satisfaction (i.e. known ceiling and floor effects) nowadays [16]. One of the advantages of FJS-12 is that it has low ceiling and floor effects [3, 17]. FJS-12 is also found to be the most responsive tool comparing with the PRO mentioned above in patients following total knee arthroplasty (TKA) [18]. FJS-12 is developed to assess the outcomes of hip and knee arthroplasty by evaluating a patient’s awareness of the artificial joint during twelve activities of daily living. FJS-12 is based upon the assumption that the goal of total knee arthroplasty is a joint patient can “forget” about. Studies started using FJS-12 as the sole PRO assessment tool [19, 20] to access knee functions and used to assess the long-term results after TKA [21].

FJS-12 constructs for shoulder, knee and hip joints and the respective questionnaire names following the joint types—FJS-12 Shoulder, FJS-12 Knee, and FJS-12 Hip. The original version of FJS-12 shows good reliability and validity [3, 22, 23]. Different language adapted versions of FJS-12 are available, including Chinese (China), Chinese (Hong Kong), and Chinese (Taiwan) versions.

World Health Organization (WHO) developed a universal measuring tool of the quality of life (QOL) called the WHOQOL Questionnaire, and WHOQOL had been translated to different languages, including Chinese (China), Chinese (Hong Kong), and Chinese (Taiwan). The development process teams of WHOQOL from mainland China, Hong Kong and Taiwan looked for the similarities and differences among these 3 language versions [24]. The authors found that, although “Chinese” language in the three regions used a similar written and spoken language and was deeply influenced by the same ancient Chinese philosophies, variations still found. The report mentioned that the differences could be attributed to a combination of historical and geo-political factors [24]. Similarities and dissimilarities can be found within subcultures [24]. The similarities and dissimilarities can also be found in other well recognized QOL measures e.g., Short Form-36 (SF-36) (SF-36 has China, Hong Kong, and Taiwan versions). Another example of sub-culture difference is also referred to the development of WHOQOL, of which WHOQOL developed USA (American English), Canadian (Canadian English), UK (British English), and Australia (Australian English) versions. That also reflects subcultural differences exist among English speaking countries.

Why is FJS-12 necessary to have the “Traditional Chinese-Hong Kong” version when “Simplified Chinese-Mandarin Chinese” version and “Traditional Chinese-Taiwan” version are available? To recall, FJS-12 has already been translated to Simplified Chinese-Mandarin Chinese [25], and translated and linguistically validated to Traditional Chinese-Taiwan [26]. “Simplified Chinese” is officially used in mainland China, Singapore and the Chinese community in Malaysia and “Traditional Chinese” is officially and commonly used in Taiwan, Hong Kong, and Macau. In the “Traditional Chinese” societies, however, a fundamental cross-cultural difference between Taiwan and Hong Kong/Macau was reported. In a cross-society comparison of general happiness and personal life satisfaction between 1222 participants from Taiwan and 1044 participants from Hong Kong using an identical survey platform, Hong Kong participants indicated a happier attitude regarding to their recent life than the Taiwanese participants [27]. However, the Taiwanese respondents were more satisfied with their personal quality of life than the Hong Kong respondents. As a result, a Traditional Chinese-Hong Kong version of FJS-12 is necessary to develop although another two Chinese versions is available now.

The purpose of this study is to validate the psychometric properties of FJS-12 by testing the reliability, validity, and responsiveness of the validated FJS-12. Floor and ceiling effects of the translated version were discussed. Oxford Knee Score (OKS) and Numeric Rating Scale (NRS) were conducted in line with the Traditional Chinese-Hong Kong version of FJS-12 and correlations between OKS and FJS-12, and between OKS and NRS were sorted.

## Methods

Between September 2019 and March 2020, 75 patients who underwent unilateral total knee arthroplasty (TKA) at their end stage of knee osteoarthritis were invited to join this study. The inclusion criteria were 1) male and female patients of any age, 2) presence of unilateral knee osteoarthritis (Kellgren Lawrence scale of III-IV), 3) patients received unilateral total knee arthroplasty at least 1 year before this study, and 4) fluent in Chinese Cantonese reading and comprehension. The exclusion criteria were 1) patients with impaired cognitive function, 2) unable to understand Chinese Cantonese, and 3) unable to self-administer both questionnaires. Informed consent was signed by every participant. Ethics approval was received from the institutional ethics review committee (ethics approval number: 2019.337). The study was performed in accordance with the Declaration of Helsinki and ICH-GCP.

### Translation and cross‑cultural adaptation

The translation of the FJS-12 into Traditional Chinese-Hong Kong version was carried out using "translation and back-translation" method, in accordance with the International Quality of Life Assessment (IQOLA) guideline [28, 29]. Following the guideline, the FJS-12 was translated from English to Traditional Chinese-Hong Kong by two independent bilingual medical professionals and one non-health worker. The translated version was then back-translated to English by two different independent bilingual medical professionals and another non-health worker. The final version was reviewed and discussed for consistency by all 6 members and subsequently verified (Version 1.1, Appendix 1). Minor modifications were made in different questions for cultural adaptation. The "modifications" were summarized in Appendix 2. "Modifications" concerned about the wordings on the same activities and actions used in different regions, and the changes were meant not to alter the meaning of the questions.

### Forgotten Joint Score-12 (FJS-12)

FJS-12 comprises 12 questions under a 5-point Likert scale (Score = 1 (never, leftmost) to 5 (mostly, rightmost)). The final score is transformed to a 0–100 scale and then reversed to obtain the final score. Higher score indicates better outcome. Scoring FJS-12 final score follows the recommended scoring algorithm.

### Oxford Knee Score (OKS)

OKS has a similar scoring algorithm with FJS-12. OKS consists of 12 assessment questions concerning pain and function after TKA scoring from 0 to 4 (0 being the worst effect and 4 being the best) [13, 30]. Summing up all 12 scores forms the final score, of which the final score ranges from 0 (most severe symptoms) to 48 (least symptoms). In recent cross-cultural adaptation and translation studies on OKS, different translated languages showed good reliability, validity and responsiveness e.g. Arabic [31], Slovenian [32], and Malaysian Chinese, Hong Kong Chinese and Singaporean Chinese [33]. The Hong Kong Traditional Chinese version of OKS was used in this study.

### Numeric Rating Scale (NRS)

NRS has been routinely applied to let the patients rate the pain level on a defined scale. NRS is a single 11-point numeric scale ranging between 0 and 10, with 0 representing “no pain” and 10 representing the pain extreme [34].

### Data collection

Validated FJS-12 and OKS was administered to the patients during their routine clinic follow-up visits (baseline). NRS was routinely recorded at each patient visit. All patients were invited to come back to the clinic 1–2 weeks after to complete these questionnaires again (follow-up).

Patients’ baseline demographics e.g., age, sex, body height, body weight, and side of surgery were collected from electronic medical records from the hospital. Details on education level of patients were not routinely collected, however, obesity in terms of body mass index (BMI) was found to be inversely associated with education level [35].

### Statistical analysis

Demographic characteristics were summarized by mean ± standard deviation (SD) for numeric data and N(%) for categorical data respectively. Reliability was measured through test–retest reliability expressed in terms of intra-class correlation (ICC) (two-way random single measure), internal consistency using Cronbach’s Alpha, and smallest detectable change (SDC) [36]. SDC was calculated using the formula: SDC = SEM × 1.96 × $\sqrt{2}$, where SEM (standard error of mean) = SD [37]. Bland–Altman plot was used to look for test–retest agreement. Correlations between FJS-12 and OKS, and between FJS-12 and NRS were tested to look for the validity between the translated version to a gold standard (construct validity). Responsiveness measuring the measurement error in longitudinal validity under repeated measures was calculated by comparing SDC with minimal important change (MIC). Floor and ceiling effects defined as the percentages of participants scoring the leftmost option “never” (“Floor”; score = 1) and rightmost option “mostly” (“Ceiling”; score = 5) in individual questions. Percentages at or above 15% considered significant [37]. Discriminant validity was evaluated using correlations between FJS-12 final score and patients’ baseline demographics. Data analysis were carried out using IBM SPSS 27.0 (Armonk, New York). A two-sided p value ≤ 0.05 was considered statistically significant.

### Bootstrapping

Bootstrapping was introduced to compare the differences in responsiveness estimates between the measures, and the results were expressed in terms of bias, standard error, and 95% confidence interval (CI) [38]. Bootstrapping is a resampling technique to draw numerous samples from the original sample with replacement [39]. In this study, a bias-corrected bootstrap method (bias corrected accelerated, BCa) with 200 and 1000 iterations or samples was used to compare the differences in the mentioned responsiveness estimates (In our study, bias, standard error, and 95% confidence interval (CI) were reported) between the measures [40–42]. Two sampling sizes, 200 and 1000 were performed because 1) this was a statistics “rule of thumb” that 200 samples provide adequate statistical power for data analysis, and 2) 1000 is a presumed sample size for running bootstrapping. Bootstrapping was also carried out using IBM SPSS 27 (Armonk, New York).

## Results

The baseline demographics of the 75 patients were tabulated in Table 1. Of the 75 patients, 74.6% were obese. Mean number of days between the baseline and follow-up was 9.53 days. Obese patients constituted 70.67% of the 75 patients, 16% were overweight and 12% felt into normal BMI range.

**Table 1:** Baseline demographics of the 75 patients underwent total knee arthroplasty

Baseline demographics	Mean ± SD (Range) or N(%)
Body height (m)	1.56 ± 0.08 (1.37, 1.76)
Body weight (kg)	66.70 ± 10.31 (51.0, 93.3)
BMI (kg/m^2^)	27.48 ± 4.22 (19.66, 37.24)
BMI	
Normal	12 (16.0)
Overweight	7 (9.4)
Obese	56 (74.6)

BMI (Asian Standards) were used according to the WHO/IASO/IOTF. The Asia–Pacific perspective: redefining obesity and its treatment. Health communication Australia Pty Ltd; 2000. Where BMI below 18.5 is underweight; from 18.5–22.9 is normal; from 23- 24.9 is overweight; from 25–34.9 is obese Where BMI below 18.5 is underweight; from 18.5–22.9 is normal; from 23–24.9 is overweight; above 25 is obese

*SD* Standard deviation, *BMI* Body Mass Index

### Reliability

**Fig. 1:**
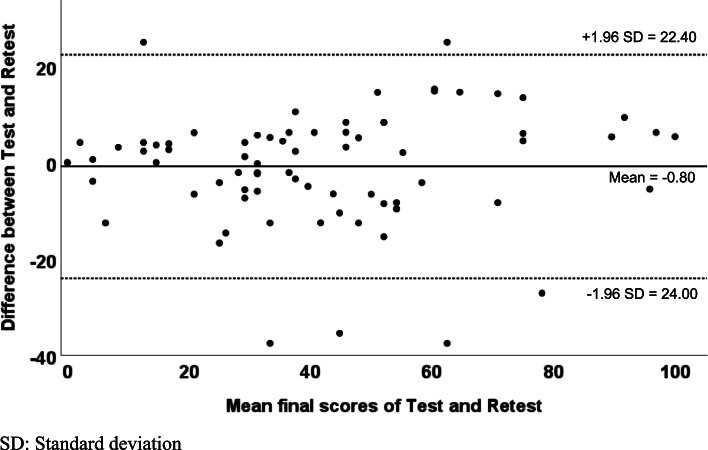
The Bland–Altman plot for test–retest (baseline—follow-up) agreement of FJS-12.

FJS-12 showed moderate to excellent internal consistency in individual question with Cronbach’s α of 0.870 in the final score (Table 2). The test–retest reliability in terms of ICC was good in the FJS-12 final score (ICC = 0.769 (95% CI = 0.560, 0.886)) using the definitions established by Koo et al. [43]. Question 1 was “excellent” and most of the questions indicated at least “moderate”. Bland–Altman plot for the repeated measures (follow-up – baseline) showed the majority of measurement differences fell within the mean ± 1.96 standard deviation (Fig. 1). Nearly all measurement differences fell within the 95% limits of agreement (LOA) (Fig. 1).

**Table 2:** Test–retest reliability and internal consistency of FJS-12 question scores between baseline and follow-up

FJS-12 questions	ICC (95% CI)	Cronbach’s α
Q1	0.832 (0.669 – 0.919)	0.908
Q2	0.734 (0.503 – 0.868)	0.847
Q3	0.517 (0.184 – 0.743)	0.681
Q4	0.651 (0.373 – 0.822)	0.789
Q5	0.720 (0.500 – 0.847)	0.720
Q6	0.734 (0.416 – 0.879)	0.734
Q7	0.745 (0.440 – 0.884)	0.745
Q8	0.666 (0.278 – 0.845)	0.666
Q9	0.788 (0.535 – 0.903)	0.788
Q10	0.520 (0.191 – 0.760)	0.520
Q11	0.839 (0.647 – 0.927)	0.839
Q12	0.755 (0.470 – 0.886)	0.755
Final score	0.769 (0.560 – 0.886)	0.870

*FJS-12* Forgotten Joint Knee Score, *ICC* Intraclass correlation (Single measure), *CI* Confidence interval

### Construct validity

Construct validity explained by correlation analyses showed moderate correlation with OKS at baseline (FJS-12 baseline vs. OKS baseline; Pearson’s coefficient = 0.598, *p* < 0.01) and very strong correlation at follow-up (Pearson’s coefficient = 0.879, *p* < 0.01) (Table 3). Similar results were also observed in correlations between FJS-12 and NRS (moderate at baseline and very strong correlation at follow-up) (Table 4).

**Table 3:** Correlations between FJS-12 final scores and OKS overall scores at baseline and follow-up

FJS-12 or OKS	FJS-12 baseline	FJS-12 follow-up	OKS baseline	OKS follow-up
FJS-12 baseline		0.771^*^^*^	0.598^**^	0.786^*^^*^
FJS-12 follow-up	0.771^*^^*^		-0.734^*^^*^	0.879^**^
OKS baseline	0.598^**^	-0.734^**^		-0.848^**^
OKS follow-up	0.786^*^^*^	0.879^**^	-0.848^**^	

*FJS*-*12* Forgotten Joint Knee Score-12 final scores, *OKS* Oxford Knee Score: overall scores

^**^ *p* < 0.01

### Responsiveness

Responsiveness in terms of SDC was 15.77. MIC was calculated by halving the standard deviation proposed by Norman et al.[44]. MIC came out to be 5.92, which was smaller than SDC (i.e., SDC was higher than MIC). Floor effect was not observable in all questions (Table 5). Ceiling effect was statistically significant in question 8 in both baseline and follow-up, unless otherwise non-specified.

**Table 4:** Correlations between FJS-12 final scores and NRS at baseline and follow-up

FJS-12 or NRS	FJS-12 baseline	FJS-12 follow-up	NRS baseline	NRS follow-up
FJS-12 baseline		0.721^*^^*^	0.601^**^	0.799^*^^*^
FJS-12 follow-up	0.721^*^^*^		-0.765^*^^*^	0.873^**^
NRS baseline	0.601^**^	-0.765^**^		-0.837^**^
NRS follow-up	0.799^*^^*^	0.873^**^	-0.837^**^	

*FJS-12* Forgotten Joint Knee Score-12 final scores, *NRS* Numeric Rating Scale

^**^ *p* < 0.01

### Discriminant validity

FJS-12 baseline and follow-up were found to have no significant correlation with patients’ age, sex, BMI, and side of surgery (Table 5). OKS baseline and follow-up were also put in line with the analysis and results also showed no significant correlation with the respective baseline demographics.

**Table 5:** Percentages of floor and ceiling effects in individual Chinese Cantonese (Hong Kong) translated FJS-12 questions collected at baseline and follow-up

Questions	Time point	Mean ± SD; Median	Floor percentage (scored “mostly”; %)	Ceiling percentage (scored “never”; %)
Q1	Baseline	3.15 ± 1.19; 3.00	14.7	12.0
	Follow-up	3.43 ± 1.14; 4.00	10.7	14.3
Q2	Baseline	3.13 ± 1.26; 3.00	14.7	12.0
	Follow-up	3.18 ± 1.25; 3.00	14.3	14.3
Q3	Baseline	3.29 ± 1.39; 4.00	13.7	24.7
	Follow-up	3.04 ± 1.37; 3.00	17.9	17.9
Q4	Baseline	2.63 ± 1.46; 2.00	32.0	13.3
	Follow-up	2.93 ± 1.44; 3.00	25.0	17.9
Q5	Baseline	3.06 ± 1.39; 3.00	20.8	15.3
	Follow-up	3.29 ± 1.21; 3.50	10.7	14.3
Q6	Baseline	3.92 ± 1.13; 4.00	6.8	32.9
	Follow-up	3.96 ± 1.00; 4.00	3.6	35.7
Q7	Baseline	3.37 ± 1.34; 4.00	15.1	23.3
	Follow-up	3.43 ± 1.17; 4.00	10.7	10.7
Q8	Baseline	3.88 ± 1.13; 4.00	6.7	32.0^*^
	Follow-up	4.18 ± 0.95; 4.00	3.6	42.9^*^
Q9	Baseline	3.60 ± 1.37; 4.00	13.7	31.5
	Follow-up	3.82 ± 1.06; 4.00	7.1	21.4
Q10	Baseline	3.53 ± 1.17; 4.00	8.2	20.5
	Follow-up	3.54 ± 1.11; 4.00	10.7	14.3
Q11	Baseline	3.25 ± 1.25; 3.00	13.7	16.4
	Follow-up	3.36 ± 1.16; 4.00	10.7	10.7
Q12	Baseline	3.33 ± 1.32; 4.00	14.7	21.3
	Follow-up	3.50 ± 1.17; 4.00	7.1	17.9

^*^ Comparisons with statistical difference, *p* < 0.01

### Bootstrapping

Bias and standard error of the mean and standard deviation of individual questions as well as total score at baseline and follow-up were both low after performing bootstrapping for 200 samples (Table 6). Similar results (low bias and standard error) were found after performing bootstrapping for 1000 samples (Table 6). In OKS, bias and standard error were low similar to that in FJS-12 (Table 7). Table 8 showed the results of mean differences, correlation coefficients, and p values in FJS-12 and OKS after bootstrapping for 200 and 1000 samples. The calculations were based on the score differences between baseline and follow-up. In mean difference, the 95% CI after bootstrapping for 200 and 1000 samples were similar (for example, in the comparison of mean difference in FJS-12 Question 1 between baseline and follow-up: within -0.46 and 0.00 in bootstrapping *N* = 200, and within -0.42 and 0.00 in bootstrapping *N* = 1000). Similarly, the 95% CI of correlation coefficients after bootstrapping *N* = 200 and N = 1000 were similar (Table 8; FJS-12 Question 1 Baseline – FJS-12 Question 1 Follow-up; bootstrapping for *N* = 200: 0.73 – 0.95; bootstrapping for *N* = 1000: 0.70 – 0.95), in both FJS-12 and OKS. The *p* values without bootstrapping and bootstrapping for *N* = 200 and *N* = 1000 were similar. The *p* values showing non-statistical significance (*p* > 0.05) without bootstrapping remained statistical insignificance after bootstrapping of both sampling sizes. In the comparison group “OKS Question 12 Baseline – OKS Question 12 Follow-up”, the score difference was found to have statistical significance (*p* = 0.05). Statistical difference remained after the two bootstrapping methods (*p* = 0.02 after bootstrapping for *N* = 200; and *p* = 0.05 after bootstrapping for *N* = 1000).

**Table 6:** Correlation between FJS-12 final scores and participants’ characteristics, and between OKS and participants’ characteristics

	Age	Sex	BMI	Side of surgery
FJS-12 (baseline)	0.160	-0.153	-0.086	-0.018
FJS-12 (follow-up)	0.099	-0.153	-0.180	-0.105
OKS (baseline)	0.097	-0.154	0.113	0.120
OKS (follow-up)	0.119	-0.080	-0.325	-0.043

*BMI* Body Mass Index, *FJS* Forgotten joint score, *OKS* Oxford Knee Score

**Table 7:** Means and standard deviations of FJS-12 individual questionnaires and total scores at baseline and follow-up, and bias, its standard error and 95% confidence intervals after bootstrapping for *N* = 200 and *N* = 1000

Questionnaire and timepoint	Mean or Standard deviation	Bootstrapping *N* = 200				Bootstrapping *N* = 1000			
		This study	Bias	Standard error	95% CI	This study	Bias	Standard error	95% CI
FJS-12 Q01 Baseline	Mean	3.17	-0.02	0.24	2.75—3.50	3.17	0.01	0.22	2.75—3.58
	Standard deviation	1.17	-0.00	0.22	0.88—1.43	1.17	-0.03	0.16	0.88—1.39
FJS-12 Q01 Follow-up	Mean	3.38	-0.03	0.22	3.00—3.67	3.38	0.01	0.23	2.92—3.79
	Standard deviation	1.17	-0.02	0.15	0.88—1.41	1.17	-0.04	0.17	0.84—1.38
FJS-12 Q02 Baseline	Mean	3.33	0.01	0.25	2.88—3.87	3.33	0.01	0.24	2.88—3.79
	Standard deviation	1.20	-0.05	0.15	0.93—1.37	1.20	-0.04	0.14	0.98—1.34
FJS-12 Q02 Follow-up	Mean	3.25	0.00	0.26	2.79—3.67	3.25	0.01	0.25	2.75—3.75
	Standard deviation	1.26	-0.05	0.15	1.01—1.42	1.26	-0.04	0.14	1.01—1.41
FJS-12 Q03 Baseline	Mean	3.29	-0.02	0.28	2.75—3.72	3.29	0.01	0.27	2.75—3.79
	Standard deviation	1.37	-0.04	0.13	1.20—1.47	1.37	-0.04	0.13	1.14—1.52
FJS-12 Q03 Follow-up	Mean	3.13	0.00	0.28	2.67—3.62	3.13	0.01	0.27	2.63—3.67
	Standard deviation	1.39	-0.04	0.14	1.19—1.51	1.39	-0.04	0.13	1.16—1.54
FJS-12 Q04 Baseline	Mean	2.63	0.00	0.29	2.17—3.17	2.63	0.01	0.28	2.04—3.13
	Standard deviation	1.44	-0.02	0.10	1.23—1.59	1.44	-0.03	0.12	1.25—1.58
FJS-12 Q04 Follow-up	Mean	3.00	0.03	0.29	2.37—3.59	3.00	0.02	0.30	2.38—3.63
	Standard deviation	1.47	-0.05	0.13	1.30—1.55	1.47	-0.04	0.14	1.27—1.60
FJS-12 Q05 Baseline	Mean	2.96	0.01	0.27	2.46—3.46	2.96	0.02	0.27	2.42—3.50
	Standard deviation	1.40	-0.03	0.13	1.14—1.56	1.40	-0.03	0.13	1.18—1.53
FJS-12 Q05 Follow-up	Mean	3.21	-0.01	0.24	2.79—3.67	3.21	0.01	0.24	2.79—3.63
	Standard deviation	1.22	-0.04	0.14	0.97—1.38	1.22	-0.03	0.14	0.98—1.38
FJS-12 Q06 Baseline	Mean	3.88	0.00	0.23	3.50—4.25	3.88	0.01	0.23	3.42—4.33
	Standard deviation	1.19	-0.04	0.20	0.78—1.47	1.19	-0.05	0.20	0.81—1.44
FJS-12 Q06 Follow-up	Mean	3.92	-0.02	0.18	3.67—4.17	3.92	0.00	0.20	3.54—4.29
	Standard deviation	1.02	-0.03	0.15	0.76—1.24	1.02	-0.04	0.17	0.75—1.23
FJS-12 Q07 Baseline	Mean	3.25	0.01	0.24	2.88—3.75	3.25	0.01	0.24	2.79—3.67
	Standard deviation	1.19	-0.03	0.14	0.96—1.35	1.19	-0.04	0.15	0.92—1.36
FJS-12 Q07 Follow-up	Mean	3.54	0.02	0.24	3.06—4.03	3.54	0.01	0.23	3.04—4.00
	Standard deviation	1.18	-0.07	0.21	0.75—1.40	1.18	-0.05	0.20	0.83—1.38
FJS-12 Q08 Baseline	Mean	3.75	0.00	0.23	3.29—4.13	3.75	0.01	0.23	3.29—4.21
	Standard deviation	1.19	-0.04	0.17	0.86—1.44	1.19	-0.04	0.18	0.88—1.38
FJS-12 Q08 Follow-up	Mean	4.13	0.00	0.19	3.79—4.50	4.13	0.00	0.20	3.75—4.50
	Standard deviation	0.99	-0.05	0.19	0.66—1.20	0.99	-0.04	0.20	0.66—1.23
FJS-12 Q09 Baseline	Mean	3.92	0.00	0.20	3.54—4.25	3.92	0.01	0.22	3.50—4.33
	Standard deviation	1.10	-0.02	0.17	0.76—1.35	1.10	-0.04	0.18	0.76—1.34
FJS-12 Q09 Follow-up	Mean	4.00	0.00	0.16	3.74—4.25	4.00	0.00	0.18	3.67—4.29
	Standard deviation	0.89	-0.04	0.20	0.55—1.12	0.89	-0.04	0.22	0.54—1.14
FJS-12 Q10 Baseline	Mean	3.63	-0.01	0.20	3.29—3.96	3.63	0.01	0.22	3.17—4.08
	Standard deviation	1.14	-0.04	0.17	0.79—1.37	1.14	-0.04	0.18	0.83—1.34
FJS-12 Q10 Follow-up	Mean	3.58	0.01	0.19	3.17—3.92	3.58	0.00	0.21	3.21—3.96
	Standard deviation	1.06	-0.06	0.18	0.65—1.26	1.06	-0.04	0.19	0.70—1.29
FJS-12 Q11 Baseline	Mean	3.33	-0.01	0.20	3.00—3.67	3.33	0.01	0.22	2.92—3.75
	Standard deviation	1.09	-0.03	0.15	0.78—1.31	1.09	-0.04	0.15	0.83—1.25
FJS-12 Q11 Follow-up	Mean	3.50	0.01	0.21	3.13—3.88	3.50	0.01	0.22	3.08—3.92
	Standard deviation	1.10	-0.05	0.17	0.76—1.29	1.10	-0.05	0.17	0.85—1.25
FJS-12 Q12 Baseline	Mean	3.29	0.00	0.20	2.98—3.67	3.29	0.01	0.22	2.88—3.71
	Standard deviation	1.08	-0.03	0.15	0.78—1.32	1.08	-0.04	0.15	0.83—1.25
FJS-12 Q12 Follow-up	Mean	3.50	0.02	0.24	3.00—4.02	3.50	0.02	0.25	2.97—4.04
	Standard deviation	1.25	-0.05	0.15	1.00—1.40	1.25	-0.04	0.15	1.03—1.38
FJS-12 Total Baseline	Mean	40.80	0.06	4.99	29.55—50.70	40.80	-0.31	4.91	32.47—48.70
	Standard deviation	25.28	-0.56	3.32	18.26—30.57	25.28	-0.76	3.36	19.54—29.29
FJS-12 Total Follow-up	Mean	37.24	-0.06	4.46	29.13—46.58	37.24	-0.23	4.68	28.65—45.58
	Standard deviation	23.63	-1.00	3.51	17.08—27.17	23.63	-0.75	3.70	17.55—28.08

**Table 8:** Means and standard deviations of OKS individual questions and total scores at baseline and follow-up, and bias, its standard error and 95% confidence intervals after bootstrapping for* N* = 200 and *N* = 1000

Question and timepoint	Mean or Standard deviation	Bootstrapping *N* = 200				Bootstrapping *N* = 1000			
		This study	Bias	Standard error	95% CI	This study	Bias	Standard error	95% CI
OKS Q01 Baseline	Mean	2.22	0.03	0.23	1.81—2.67	2.22	0.00	0.23	1.81—2.63
	Standard deviation	1.22	-0.02	0.12	0.99—1.37	1.22	-0.02	0.14	0.94—1.42
OKS Q01 Follow-up	Mean	2.19	0.03	0.23	1.81—2.63	2.19	0.00	0.23	1.81—2.56
	Standard deviation	1.21	-0.02	0.11	1.04—1.35	1.21	-0.02	0.13	1.00—1.36
OKS Q02 Baseline	Mean	3.07	0.01	0.17	2.78—3.38	3.07	-0.01	0.16	2.81—3.33
	Standard deviation	0.83	-0.03	0.11	0.68—0.94	0.83	-0.02	0.11	0.65—0.96
OKS Q02 Follow-up	Mean	3.00	0.00	0.18	2.70—3.30	3.00	0.00	0.18	2.67—3.26
	Standard deviation	0.92	-0.02	0.17	0.62—1.18	0.92	-0.04	0.18	0.63—1.14
OKS Q03 Baseline	Mean	2.78	0.01	0.18	2.46—3.10	2.78	0.00	0.17	2.52—3.07
	Standard deviation	0.89	-0.02	0.09	0.75—1.00	0.89	-0.02	0.10	0.71—1.01
OKS Q03 Follow-up	Mean	2.74	0.01	0.20	2.40—3.12	2.74	-0.01	0.18	2.44—3.04
	Standard deviation	0.94	-0.02	0.10	0.79—1.07	0.94	-0.02	0.09	0.80—1.05
OKS Q04 Baseline	Mean	3.33	0.02	0.18	2.96—3.72	3.33	0.00	0.18	3.04—3.59
	Standard deviation	0.92	-0.05	0.12	0.70—1.01	0.92	-0.02	0.12	0.69—1.10
OKS Q04 Follow-up	Mean	3.26	0.02	0.14	3.00—3.59	3.26	0.00	0.16	3.00—3.52
	Standard deviation	0.81	-0.02	0.08	0.69—0.89	0.81	-0.02	0.07	0.68—0.90
OKS Q05 Baseline	Mean	2.89	0.02	0.17	2.59—3.27	2.89	0.00	0.16	2.63—3.15
	Standard deviation	0.85	-0.03	0.09	0.71—0.93	0.85	-0.02	0.09	0.71—0.94
OKS Q05 Follow-up	Mean	2.78	0.03	0.16	2.44—3.19	2.78	0.00	0.17	2.48—3.04
	Standard deviation	0.89	-0.03	0.11	0.71—1.01	0.89	-0.02	0.12	0.65—1.05
OKS Q06 Baseline	Mean	2.78	0.02	0.24	2.33—3.27	2.78	0.00	0.23	2.44—3.15
	Standard deviation	1.22	-0.03	0.14	0.96—1.41	1.22	-0.03	0.14	0.92—1.42
OKS Q06 Follow-up	Mean	2.74	0.01	0.22	2.36—3.15	2.74	0.00	0.22	2.37—3.11
	Standard deviation	1.23	-0.02	0.16	0.89—1.50	1.23	-0.02	0.16	0.88—1.48
OKS Q07 Baseline	Mean	1.22	0.01	0.28	0.63—1.82	1.22	0.00	0.27	0.70—1.76
	Standard deviation	1.42	-0.02	0.14	1.16—1.64	1.42	-0.03	0.15	1.16—1.60
OKS Q07 Follow-up	Mean	1.07	0.01	0.27	0.58—1.64	1.07	0.00	0.28	0.63—1.59
	Standard deviation	1.44	-0.02	0.15	1.11—1.66	1.44	-0.04	0.16	1.14—1.62
OKS Q08 Baseline	Mean	2.85	0.01	0.21	2.48—3.24	2.85	0.00	0.21	2.52—3.19
	Standard deviation	1.10	-0.03	0.13	0.86—1.23	1.10	-0.02	0.12	0.88—1.25
OKS Q08 Follow-up	Mean	2.74	0.03	0.22	2.33—3.23	2.74	-0.01	0.23	2.30—3.11
	Standard deviation	1.20	-0.04	0.14	0.95—1.36	1.20	-0.02	0.13	0.94—1.39
OKS Q09 Baseline	Mean	2.52	0.02	0.19	2.19—2.89	2.52	0.00	0.18	2.22—2.81
	Standard deviation	0.98	-0.02	0.09	0.81—1.08	0.98	-0.02	0.11	0.82—1.09
OKS Q09 Follow-up	Mean	2.52	0.01	0.18	2.19—2.86	2.52	-0.01	0.18	2.22—2.78
	Standard deviation	0.94	-0.03	0.14	0.64—1.12	0.94	-0.03	0.14	0.68—1.14
OKS Q10 Baseline	Mean	2.93	0.00	0.18	2.67—3.26	2.93	0.00	0.18	2.63—3.22
	Standard deviation	0.96	-0.02	0.12	0.70—1.13	0.96	-0.02	0.12	0.71—1.13
OKS Q10 Follow-up	Mean	3.00	0.01	0.17	2.70—3.30	3.00	0.01	0.17	2.70—3.30
	Standard deviation	0.88	-0.03	0.13	0.64—1.03	0.88	-0.02	0.12	0.65—1.04
OKS Q11 Baseline	Mean	2.93	0.01	0.27	2.37—3.43	2.93	-0.01	0.23	2.52—3.30
	Standard deviation	1.21	-0.04	0.21	0.86—1.44	1.21	-0.03	0.18	0.86—1.45
OKS Q11 Follow-up	Mean	2.85	0.01	0.22	2.48—3.27	2.85	0.00	0.20	2.52—3.19
	Standard deviation	1.06	-0.03	0.11	0.83—1.19	1.06	-0.02	0.10	0.90—1.17
OKS Q12 Baseline	Mean	2.85	0.01	0.19	2.44—3.22	2.85	0.00	0.19	2.53—3.15
	Standard deviation	0.99	-0.03	0.17	0.70—1.25	0.99	-0.03	0.16	0.71—1.22
OKS Q12 Follow-up	Mean	2.44	0.02	0.25	1.96—3.02	2.44	0.00	0.23	2.04—2.85
	Standard deviation	1.25	-0.03	0.14	1.05—1.40	1.25	-0.04	0.15	1.00—1.42
OKS Total Baseline	Mean	27.11	-0.13	1.93	23.59—30.23	27.11	0.02	1.82	23.33—30.91
	Standard deviation	9.64	-0.17	0.94	7.98—10.78	9.64	-0.21	0.95	7.86—10.90
OKS Total Follow-up	Mean	31.33	0.20	1.95	27.52—35.73	31.33	-0.03	1.90	27.87—34.59
	Standard deviation	9.96	-0.17	0.89	8.17—11.31	9.96	-0.21	1.01	8.05—11.27

Cross-comparisons between FJS-12 and OKS individual scores at baseline and follow-up followed. In the comparisons between FJS-12 and OKS in the 13 individual questions (12 questions and total) at baseline, mean differences and correlation coefficients were similar (Table 9). These results were reflected by the p values without bootstrapping, bootstrapping for* N* = 200, and bootstrapping for *N* = 1000 (Table 10). Comparing between 95% CI of mean difference and 95% CI of correlation coefficient in FJS-12 and OKS after bootstrapping for *N* = 200 and for *N *= 100 showed similar results (Table 11). For example, in the comparison “FJS-12 Q01 Follow-up – OKS Q01 Follow-up”, the 95% CI of mean differences were 0.43 to 2.14 (bootstrapping for *N* = 200) and 0.43 to 2.14 (bootstrapping for *N* = 1000) (Table 11, first row). The *p* values were 0.01 (without bootstrapping), 0.02 (bootstrapping for *N* = 200), and 0.01 (bootstrapping for *N* = 1000). Comparisons showing statistical significance (i.e. *p* < 0.05) without applying bootstrapping remained statistically significant after bootstrapping for *N* = 200 and for *N* = 1000. This was reflected in comparisons “FJS-12 Q06 Follow-up – OKS Q06 Follow-up”, “FJS-12 Q07 Follow-up – OKS Q07 Follow-up”, “FJS-12 Q08 Follow-up – OKS Q08 Follow-up”, “FJS-12 Q09 Follow-up – OKS Q09 Follow-up”, “FJS-12 Q12 Follow-up – OKS Q12 Follow-up”,

**Table 9:** Summary table of the 95% CI of mean difference, correlation coefficient, and p value between FJS-12 baseline and FJS-12 follow-up in individual questions using paired T-tests after applying bootstrapping with *N* = 200 and *N* = 1000

Question and timepoint		Bootstrapping *N* = 200	Bootstrapping *N* = 1000		Bootstrapping *N* = 200	Bootstrapping *N* = 1000		Bootstrapping *N* = 200	Bootstrapping *N* = 1000
	Mean difference	95% CI of mean difference after bootstrap	95% CI of mean difference after bootstrap	Correlation coefficient	95% CI of correlation coefficient after bootstrap	95% CI of correlation coefficient after bootstrap	*P* value	*P* value after bootstrap	*P *value after bootstrap
FJS-12 Q01 Baseline – FJS-12 Q01 Follow-up	-0.21	-0.46—0.00	-0.42—0.00	0.87	0.73—0.95	0.70—0.95	0.10	0.10	0.09
FJS-12 Q02 Baseline – FJS-12 Q02 Follow-up	0.08	-0.19—0.36	-0.13—0.29	0.86	0.73—0.93	0.70—0.94	0.54	0.51	0.52
FJS-12 Q03 Baseline – FJS-12 Q03 Follow-up	0.17	-0.34—0.68	-0.25—0.58	0.62	0.26—0.87	0.28—0.83	0.50	0.48	0.49
FJS-12 Q04 Baseline – FJS-12 Q04 Follow-up	-0.38	-0.90—0.15	-0.83—0.00	0.64	0.34—0.86	0.25—0.86	0.15	0.17	0.16
FJS-12 Q05 Baseline – FJS-12 Q05 Follow-up	-0.25	-0.88—0.38	-0.83—0.29	0.36	-0.11—0.72	-0.08—0.74	0.42	0.41	0.42
FJS-12 Q06 Baseline – FJS-12 Q06 Follow-up	-0.04	-0.46—0.38	-0.46—0.33	0.60	0.00—0.95	-0.12—0.96	0.84	0.88	0.85
FJS-12 Q07 Baseline – FJS-12 Q07 Follow-up	-0.29	-0.73—0.15	-0.63—0.04	0.61	0.29—0.84	0.24—0.84	0.18	0.20	0.18
FJS-12 Q08 Baseline – FJS-12 Q08 Follow-up	-0.38	-0.82—0.07	-0.79—0.00	0.54	0.05—0.89	-0.02—0.90	0.10	0.14	0.13
FJS-12 Q09 Baseline – FJS-12 Q09 Follow-up	-0.08	-0.41—0.24	-0.33—0.21	0.72	0.37—0.87	0.33—0.89	0.60	0.61	0.59
FJS-12 Q10 Baseline – FJS-12 Q10 Follow-up	0.04	-0.45—0.53	-0.38—0.46	0.44	0.00—0.82	-0.05—0.84	0.86	0.88	0.86
FJS-12 Q11 Baseline – FJS-12 Q11 Follow-up	-0.17	-0.46—0.13	-0.42—0.04	0.80	0.53—0.92	0.56—0.90	0.26	0.28	0.27
FJS-12 Q12 Baseline – FJS-12 Q12 Follow-up	-0.21	-0.62—0.20	-0.56—0.13	0.66	0.32—0.84	0.37—0.82	0.31	0.32	0.30
FJS-12 Total Baseline – FJS-12 Total Follow-up	3.26	-3.24—10.36	-2.64—10.02	0.79	0.58—0.92	0.60—0.88	0.29	0.31	0.27
OKS Q01 Baseline – OKS Q01 Follow-up	0.04	-0.11—0.19	-0.14—0.21	0.94	0.83—0.99	0.85—0.98	0.66	0.69	0.66
OKS Q02 Baseline – OKS Q02 Follow-up	0.07	-0.19—0.41	-0.22—0.36	0.66	0.36—0.81	0.46—0.82	0.60	0.57	0.59
OKS Q03 Baseline – OKS Q03 Follow-up	0.04	-0.30—0.26	-0.24—0.32	0.71	0.51—0.86	0.45—0.90	0.79	0.82	0.79
OKS Q04 Baseline – OKS Q04 Follow-up	0.07	-0.22—0.41	-0.32—0.47	0.34	-0.05—0.68	-0.05—0.68	0.70	0.67	0.70
OKS Q05 Baseline – OKS Q05 Follow-up	0.11	-0.07—0.33	-0.17—0.39	0.68	0.42—0.88	0.40—0.90	0.42	0.40	0.41
OKS Q06 Baseline – OKS Q06 Follow-up	0.04	-0.19—0.30	-0.20—0.27	0.89	0.77—0.94	0.77—0.95	0.75	0.69	0.78
OKS Q07 Baseline – OKS Q07 Follow-up	0.15	-0.24—0.56	-0.33—0.62	0.65	0.36—0.87	0.35—0.85	0.53	0.54	0.51
OKS Q08 Baseline – OKS Q08 Follow-up	0.11	-0.16—0.37	-0.19—0.41	0.79	0.64—0.89	0.54—0.93	0.45	0.46	0.46
OKS Q09 Baseline – OKS Q09 Follow-up	0.00	-0.22—0.27	-0.31—0.31	0.66	0.41—0.89	0.39—0.89	1.00	1.00	1.00
OKS Q10 Baseline – OKS Q10 Follow-up	-0.07	-0.33—0.26	-0.38—0.24	0.64	0.38—0.82	0.37—0.83	0.63	0.63	0.62
OKS Q11 Baseline – OKS Q11 Follow-up	0.07	-0.17—0.33	-0.24—0.38	0.77	0.56—0.89	0.54—0.91	0.63	0.61	0.64
OKS Q12 Baseline – OKS Q12 Follow-up	0.41	0.07—0.75	0.01—0.81	0.62	0.40—0.81	0.41—0.84	0.05	0.02	0.05
OKS Total Baseline – OKS Total Follow-up	-4.22	-10.70—2.31	-11.80—3.35	-0.91	-0.96—-0.80	-0.97—-0.79	0.26	0.24	0.27

**Table 10:** Summary table of the 95% CI of mean difference, correlation coefficient, and p value comparing the scores in individual questions and total between FJS-12 and OKS at baseline using paired T-tests applying bootstrapping with *N = *200 and *N* = 1000

Question and timepoint		Bootstrapping *N* = 200	Bootstrapping* N* = 1000		Bootstrapping *N* = 200	Bootstrapping *N* = 1000		Bootstrapping *N* = 200	Bootstrapping *N* = 1000
	Mean difference	95% CI of mean difference after bootstrap	95% CI of mean difference after bootstrap	Correlation coefficient	95% CI of correlation coefficient after bootstrap	95% CI of correlation coefficient after bootstrap	*P* value	*P *value after bootstrap	*P* value after bootstrap
FJS-12 Q01 Baseline – OKS Q01 Baseline	-0.21	-0.46—0.00	-0.42—0.00	0.87	0.73—0.95	0.71—0.94	0.10	0.10	0.09
FJS-12 Q02 Baseline – OKS Q02 Baseline	0.08	-0.19—0.36	-0.13—0.29	0.86	0.73—0.93	0.70—0.94	0.54	0.51	0.52
FJS-12 Q03 Baseline – OKS Q03 Baseline	0.17	-0.34—0.68	-0.25—0.58	0.62	0.26—0.87	0.28—0.83	0.50	0.48	0.49
FJS-12 Q04 Baseline – OKS Q04 Baseline	-0.38	-0.90—0.15	-0.83—0.00	0.64	0.34—0.86	0.25—0.86	0.15	0.17	0.16
FJS-12 Q05 Baseline – OKS Q05 Baseline	-0.25	-0.88—0.38	-0.83—0.29	0.36	-0.11—0.72	-0.08—0.74	0.42	0.41	0.42
FJS-12 Q06 Baseline – OKS Q06 Baseline	-0.04	-0.46—0.38	-0.46—0.33	0.60	0.00—0.95	-0.12—0.96	0.84	0.88	0.85
FJS-12 Q07 Baseline – OKS Q07 Baseline	-0.29	-0.73—0.15	-0.63—0.04	0.61	0.29—0.84	0.24—0.84	0.18	0.20	0.18
FJS-12 Q08 Baseline – OKS Q08 Baseline	-0.38	-0.82—0.07	-0.79—0.00	0.54	0.05—0.89	-0.02—0.90	0.10	0.14	0.13
FJS-12 Q09 Baseline – OKS Q09 Baseline	-0.08	-0.41—0.24	-0.33—0.21	0.72	0.37—0.87	0.33—0.89	0.60	0.61	0.59
FJS-12 Q10 Baseline – OKS Q10 Baseline	0.04	-0.45—0.53	-0.38—0.46	0.44	0.00—0.82	-0.05—0.84	0.86	0.88	0.86
FJS-12 Q11 Baseline – OKS Q11 Baseline	-0.17	-0.46—0.13	-0.42—0.04	0.80	0.53—0.92	0.56—0.90	0.26	0.28	0.27
FJS-12 Q12 Baseline – OKS Q12 Baseline	-0.21	-0.62—0.20	-0.56—0.13	0.66	0.32—0.84	0.37—0.82	0.31	0.32	0.30
FJS-12 Total Baseline – OKS Total Baseline	3.26	-3.24—10.36	-2.64—10.02	0.79	0.58—0.92	0.60—0.88	0.29	0.31	0.27

**Table 11:** Summary table of the 95% CI of mean difference, correlation coefficient, and p value comparing the scores in individual questions and total between FJS-12 and OKS at follow-up using paired T-tests applying bootstrapping with *N = *200 and *N* = 1000

Question and timepoint		Bootstrapping *N* = 200	Bootstrapping *N* = 1000		Bootstrapping *N* = 200	Bootstrapping *N* = 1000		Bootstrapping *N *= 200	Bootstrapping *N* = 1000
	Mean difference	95% CI of mean difference after bootstrap	95% CI of mean difference after bootstrap	Correlation coefficient	95% CI of correlation coefficient after bootstrap	95% CI of correlation coefficient after bootstrap	*P* value	*P *value after bootstrap	*P* value after bootstrap
FJS-12 Q01 Follow-up – OKS Q01 Follow-up	1.29	0.43—2.14	0.43—2.14	-0.78	-0.87—-0.65	-0.88—0.64	0.01	0.02	0.01
FJS-12 Q02 Follow-up – OKS Q02 Follow-up	0.18	-0.55—0.91	-0.55—0.91	-0.53	-0.79—-0.29	-0.81—-0.26	0.62	0.68	0.62
FJS-12 Q03 Follow-up – OKS Q03 Follow-up	0.32	-0.48—1.12	-0.48—1.12	-0.57	-0.80—-0.27	-0.79—-0.25	0.42	0.44	0.42
FJS-12 Q04 Follow-up – OKS Q04 Follow-up	-0.29	-1.03—0.46	-1.03—0.46	-0.39	-0.70—0.03	-0.67—-0.08	0.44	0.48	0.44
FJS-12 Q05 Follow-up – OKS Q05 Follow-up	0.54	-0.21—1.28	-0.21—1.28	-0.65	-0.85—-0.37	-0.84—-0.39	0.15	0.17	0.15
FJS-12 Q06 Follow-up – OKS Q06 Follow-up	1.21	0.47—1.96	0.47—1.96	-0.53	-0.75—-0.28	-0.73—-0.30	< 0.01	0.01	< 0.01
FJS-12 Q07 Follow-up – OKS Q07 Follow-up	2.39	1.52—3.27	1.52—3.27	-0.50	-0.81—0.01	-0.83 – 0.02	< 0.01	0.01	< 0.01
FJS-12 Q08 Follow-up – OKS Q08 Follow-up	1.46	0.79—2.14	0.79—2.14	-0.35	-0.60—-0.06	-0.56—-0.05	< 0.01	0.01	< 0.01
FJS-12 Q09 Follow-up – OKS Q09 Follow-up	1.36	0.69—2.03	0.69—2.03	-0.46	-0.67—-0.27	-0.63—-0.31	< 0.01	0.01	< 0.01
FJS-12 Q10 Follow-up – OKS Q10 Follow-up	0.57	-0.09—1.23	-0.09—1.23	-0.47	-0.69—-0.21	-0.69—-0.22	0.09	0.10	0.09
FJS-12 Q11 Follow-up – OKS Q11 Follow-up	0.54	-0.25—1.32	-0.25—1.32	-0.67	-0.82—-0.47	-0.83—-0.49	0.17	0.19	0.17
FJS-12 Q12 Follow-up – OKS Q12 Follow-up	1.04	0.29—1.78	0.29—1.78	-0.27	-0.58—-0.24	-0.57—0.07	0.01	0.01	0.01
FJS-12 Total Follow-up – OKS Total Follow-up	7.21	1.38—13.04	1.38—13.04	-0.88	0.80—0.94	0.79—0.95	0.02	0.03	0.02

## Discussion

This study validated the Traditional Chinese-Hong Kong version of FJS-12. The 75 patients underwent TKA for at least 1 year completed the translated FJS-12 twice, about 2 weeks apart. All patients also completed OKS at the two time points serving as the gold standard. Results showed moderate to excellent reliability and validity in FJS-12, in both individual questions and final score. Relationship between the differences in mean and mean values between baseline and follow-up showed good agreement. Responsiveness was proven fine with the absence of ceiling or floor effect. Discriminant validity showed no significant correlation between final score and baseline demographical variables.

Obesity is a well-known risk factor for OA, and end-stage OA patients demand for TKA. World Health Organization (WHO) released a brochure on "Global Strategy on Diet, Physical Activity and Health" in year 2004 [45] followed by a global action plan on physical activity 2018–2030 in year 2018 [46]. A recent report projected the obesity trend in 2030 that the number of people who are overweigh might reach a total of 2.16 billion and another 1.12 billion obese population, or 38% and 20% of the world's adult population respectively [47]. Mean BMI of patients in our previous studies always fell within “overweight” or “obese” categories [48–50]. Consequently, a PRO questionnaire for patients underwent TKA is important to provide accurate and high responsiveness to the respondents (patients) who are “overweight” or “obese”. The effect of BMI on results from different PRO questionnaires are somehow conflicting [51–53]. FJS-12 has been proven to be simple, valid and reliable in original and translated versions [3, 17, 20, 54–56]. A study in New York found that although patients who were obese (BMI ≥ 30 kg/m^2^) and received primary TKA provided lower post-surgery FJS-12 scores, statistical significance was not found [57]. That means FJS-12 is able to accurately reflect patients’ outcome undergoing conservative or operative treatment of the knee, regardless of the patient’s BMI. The mean BMI of our patients was 27.48 which was classified as “obese” (using BMI categories for Asians [58]). We speculate the percentage of obese patients would be ever increasing. The education level of our patients also reflects the necessity of having a Traditional Chinese-Hong Kong version of FJS-12 for local community. The validated FJS-12 is, therefore, suitable for any patients who linguistically prefer Traditional Chinese-Hong Kong version.

There are 3 questions which either ICC or Cronbach’s alpha was lower than 0.7. The 3 questions are: Q3. when you are walking for more than 15 min, Q8. when you are standing up from a low-sitting position, and Q10. when you are doing housework or gardening. Looking at the percentages of “floor” and “ceiling” answers in these questions can identify the causes. In question 3, 24.7% of patients were never aware of their artificial joints when walking for more than 15 min (the higher percentage of “never” means better (already forgotten their artificial joints)) and this percentage has been decreased to 17.9% after 2 weeks. Similarly, the percentage of answering “mostly” increased by 4.2% (17.9%—13.7%) meaning more patients took extra attention to their knee implants after at least 15-min walk. Patients tended not to “forget their knee implants” within the test–retest period. In this study, the period administering both questionnaires between the 2 rounds was about 2 weeks, which was similar in other validation studies [25, 55, 59]. As a result, the percentages had been changed and the changes made the ICC and Cronbach’s alpha lower comparing with other questions. Similar phenomenon was also observed in question 10 (patients were “alerted” and fewer patients forgot their knee implants when doing housework). In question 8, statistical significances were found between patients scoring “mostly” and “never” in both baseline and follow-up. Percentages of patients reflecting “never” thought of their artificial knee joints increased from 32.0% at baseline to 42.9% at follow-up, and at the same time, the percentages of patients mostly aware of their knees decreased. The results of Q8 (when you are standing up from a low-sitting position) are contrary to those of Q3 (when you are walking for more than 15 min) and Q10 (when you are doing housework or gardening) because walking for more than 15 min and doing housework or gardening are continuously performing while standing up from a low-sitting position is an example of split-second movement. Patients on artificial knee joints tend to be aware of their joints after these kinds of continuous activities over time (reflected by the decreased ceiling percentages and increased floor percentages). Patients gain confidence on short-term movements over time; therefore, more patients “forget” their artificial joint(s) when they stand up from a lower-sitting position. No significant floor and ceiling effect was observed through a recent validity study in the UK evaluating the Oxford Knee Score using a national patient-reported outcome measure dataset [60].

Correlations between translated FJS-12 and OKS are promising. We correlated FJS-12 with OKS at baseline and follow-up, and results were 0.598 at baseline and 0.879 at follow-up. In different validation studies on language adaptations using OKS as gold standard, correlation coefficients were 0.366 in German version [55] and 0.37 in Hindi version [59]. Our results showed moderate correlation when patients first answered the FJS-12 and good correlation at the repeated administration. Previous studies showed FJS-12 was more responsive at 6 months and 12 months[61], and 1 to 2 years after surgery [18]. We conclude that the responsiveness of FJS-12 is good for knee OA patients after TKA. The subjects in this study experienced TKA at least after 1 year and the responsiveness of FJS-12 was proven better 1 to 2 years after surgery [18]. Further study on inviting patients to complete FJS-12 shortly after TKA to look for the responsiveness immediately after surgery to 6 or 9 months after can fill out responsiveness data gap before 1 year after TKA. We chose OKS as the gold standard because both questionnaires share similar construct (12 questions) and total sum is calculated by simply adding all 12 scores (“final score” in FJS-12 and “overall score” in OKS; data conversion reverting the score strength in FJS-12 is require without data transformation). Both total sums can be scaled to a maximum of 100 (native in FJS-12 and ratio conversion in OKS). That would make the two questionnaires easily comparable. Furthermore, only OKS is introduced in this study which is different from other studies which employed multiple PRO tools to validate the language adapted version. The mean age of our patients was around 70 years old [49] and response bias happened when old age patients required to fill out multiple questionnaires. Telephone interview instead of face-to-face interview could have been an alternative but declined eventually because the targets were elderly patients who were prone to lower response rates [62–64] and they could cope with short interview duration only [62, 63]. Mailing all sets of questionnaires to the participants hoping them to complete and send the questionnaires back at different time points was reported low response rate. The Dutch version came with a limitation of receiving all questionnaires back after sending two sets of questionnaires in one go expecting to receive the second set within 2 weeks [65]. Further study on developing an electronic version of FJS-12 and accessing the FJS-12 through a web/mobile browser or mobile phone application could possibly increase the successful rate. Furthermore, if the electronic version can easily switch languages instantly, that will definitely increase the response rate in communities which use different kinds of official languages e.g., switch between English or French in Canada.

Using Bland–Altman (BA) plot explaining the agreement between two methods or test–retest reliability is very useful and clear to demonstrate any systematic error between the two measures. This confirms the good test–retest (baseline-follow-up) agreement and reproducibility of FJS-12. Our previous experience on the use of BA plots to evaluate the agreements between a new imaging technology to the conventional X-ray methods was proven useful [66].

Another important message we would like to bring out from this study is to raise the awareness of sub-culture difference within the same ethnicity or race. We firstly introduce this point by referencing to the experience of cultural adaptation and validation of WHOQOL questionnaire. WHOQOL had been translated to Chinese (China), Chinese (Hong Kong), and Chinese (Taiwan) languages [24]. Later, the Taiwan Chinese language adaption group published another article on testing the agreement between “Taiwan Chinese” version and “Taiwanese” version of the brief version of the WHOQOL [67]. The authors pointed out that > 50% of the elderly Taiwanese at age over 65 only used a spoken language, Taiwanese. Another classic example we mentioned before, is that WHOQOL is also available in American English, Canadian English, British English, and Australian English. We speculate that sub-culture variations happen in African countries, European countries, middle East countries, Southeast Asia countries, and possibly any countries with multicultural societies or federal multicultural policies. In summary, sub-culture difference is recommended to review and consider including in future version of IQOLA project. Further longitudinal study examining the long-term reflection of FJS-12 scores to patients underwent TKA is also recommended to look for any practical change over time.

### Limitations of this study

The small sample size in this study reduces the data generalizability and affects the accuracy and reliability of the results of this study. This study was carried out during COVID-19 pandemic and the patients were recruited when the local situation was being eased. We stuck onto the original research protocol to collect two sets of questionnaires through face-to-face interview. Moreover, we introduced bootstrapping to tackle the small sample size issue. Bootstrapping is an appropriate way to control and check the stability of the result. The estimates of standard errors and confidence intervals are both promising after bootstrapping for *N* = 200 and *N* = 1000. Second, we admit that using multiple gold standards increase the validity of the translated version. However, our experience tells us that when patients move on to the second questionnaire, they start asking questions on why the questions are similar to the first one. Some patients requested to opt out from the study. This affects the compliance rate. Therefore, we choose the well-recognized patient subjective outcome assessment (i.e., OKS) as the sole gold standard in this study. In view of this situation, NRS was added to correlate with FJS-12 although NRS might not be classified as “gold standard tool”. Minimal important change (MIC) in calculating responsiveness is an estimate which needs to establish a gold standard. MIC of Hindu version is 8.67 and 10.9 in German version. Further study on standardizing the calculation of MIC is recommended.

## Conclusions

Traditional Chinese-Hong Kong version of FJS-12 showed good reliability and validity for patients underwent TKA. The “Forgotten joint” score questionnaire did a great job to evaluate how the patients “forget” their artificial joint during their daily activities. FJS-12 is also suitable for patients who are obese (or body mass index (BMI) non-specific). Individual questions and final score did not carry any floor effect and ceiling effect. FJS-12 also found to have good agreement, nice responsiveness and discriminant validity. FJS-12 are important PRO questionnaires for patients who come across TKA with benefits outstand other PRO tools. Moreover, sub-cultural adaptation should be considered along with the standard guideline during cross-cultural adaptation and validation.

## Supplementary Information

**Additional file1 **

## Data Availability

The datasets used and/or analysed during the current study are available from the corresponding author on reasonable request.

## Abbreviations

FJS-12: Forgotten Joint Score-12
PRO: Patient-reported outcome
HRQOL: Health-related quality of life
WOMAC: Western Ontario and McMaster Universities Osteoarthritis Index
OKS: Oxford Knee Score
NRS: Numeric Rating Scale
KOOS: Knee Injury and Osteoarthritis Outcome Score
KSS: Knee Society Score
KFS: Knee Function Score
TKA: Total knee arthroplasty
FJS-12: Chinese Cantonese (Hong Kong) version of Forgotten Joint Score-12
ISPOR: The Professional Society for Health Economics and Outcomes Research
SD: Standard deviation
ICC: Intra-class correlation
SDC: Smallest detectable change
MIC: Minimal important change
LOA: Limits of agreement
BMI: Body mass index
OA: Osteoarthritis
WHO: World Health Organization
BAplot: Bland-Altmanplot
