# Supplementary material for: Traditional Chinese-Hong Kong version of Forgotten Joint Score-12 (FJS-12) for patients with osteoarthritis of the knee underwent joint replacement surgery: cross-cultural and sub-cultural adaptation, and validation
Source: BMC Musculoskelet Disord. 2022 Mar 8;23:222. doi: 10.1186/s12891-022-05156-5 (PMC8902851; doi:10.1186/s12891-022-05156-5)
Supplement: Supplementary file 1 — Additional file1 [file 12891_2022_5156_MOESM1_ESM.docx]

Appendix 1: Forgotten Joint Score-12 Traditional Chinese-Hong Kong version

Appendix 2: Changes made between Traditional Chinese-Taiwan language and Traditional Chinese-Hong Kong language versions of Forgotten Joint Score-12, with reference to the original English version

| Original English version | Traditional Chinese-Taiwan | Traditional Chinese-Hong Kong |
| --- | --- | --- |
| Scales: |  |  |
| Never | 不曾 | 從不 |
| Almost never | 幾乎不曾 | 幾乎沒有 |
| Seldom | 不常 | 很少 |
| Sometimes | 有時候 | [Same] |
| Mostly | 大部份的時候 | 大部份的時間 |
|  |  |  |
| Questions: |  |  |
| … in bed at night? | … 晚上在床上時？ | [Same] |
| … when you are sitting on a chair for more than one hour? | ... 當您坐在椅子上超過1小時？ | [Same] |
| … when you are walking for more than 15 minutes? | ... 當您走路超過15分鐘時？ | [Same] |
| … when you are taking a bath/shower? | ... 當您浸浴/淋浴時？ | [Same] |
| … when you are traveling in a car? | ... 當您坐車旅行時？ | ... 當您坐在車輛行駛時？ |
| … when you are climbing stairs? | … 當您爬樓梯時？ | … 當您行樓梯時？ |
| … when you are walking on uneven ground? | … 當您走在不平坦的地面上時？ | … 當您在不平坦的地面上行走時？ |
| … when you are standing up from a low-sitting position? | … 當您從較低的坐姿站起來時？ | [Same] |
| … when you are standing for long periods of time? | ... 當您長時間站立時？ | [Same] |
| … when you are doing housework or gardening? | … 當您做家事或園藝時？ | … 當您處理家務或園藝種植時？ |
| … when you are taking a walk/hiking? | ... 當您散步/健行時？ | [Same] |
| … when you are doing your favorite sport? | … 當您從事您最喜愛的運動時？ | … 當您進行您最喜愛的運動時？ |

[Same]: Wordings are the same as in the Traditional Chinese-Taiwan version
